# From the desktop to the grid: scalable bioinformatics via workflow conversion

**DOI:** 10.1186/s12859-016-0978-9

**Published:** 2016-03-12

**Authors:** Luis de la Garza, Johannes Veit, Andras Szolek, Marc Röttig, Stephan Aiche, Sandra Gesing, Knut Reinert, Oliver Kohlbacher

**Affiliations:** Center for Bioinformatics and Dept. of Computer Science, University of Tübingen, Sand 14, Tübingen, 72070 Germany; Algorithmic Bioinformatics, Computer Science Institute, Freie Universität Berlin, Takustr. 9, Berlin, 14195 Germany; College of Engineering, University of Notre Dame, 257 Fitzpatrick Hall, Notre Dame, 46556 IN United States

**Keywords:** Workflow, Interoperability, KNIME, Grid, Cloud, Galaxy, gUSE

## Abstract

**Background:**

Reproducibility is one of the tenets of the scientific method. Scientific experiments often comprise complex data flows, selection of adequate parameters, and analysis and visualization of intermediate and end results. Breaking down the complexity of such experiments into the joint collaboration of small, repeatable, well defined tasks, each with well defined inputs, parameters, and outputs, offers the immediate benefit of identifying bottlenecks, pinpoint sections which could benefit from parallelization, among others. Workflows rest upon the notion of splitting complex work into the joint effort of several manageable tasks.

There are several engines that give users the ability to design and execute workflows. Each engine was created to address certain problems of a specific community, therefore each one has its advantages and shortcomings. Furthermore, not all features of all workflow engines are royalty-free —an aspect that could potentially drive away members of the scientific community.

**Results:**

We have developed a set of tools that enables the scientific community to benefit from workflow interoperability. We developed a platform-free structured representation of parameters, inputs, outputs of command-line tools in so-called *Common Tool Descriptor* documents. We have also overcome the shortcomings and combined the features of two royalty-free workflow engines with a substantial user community: the *Konstanz Information Miner*, an engine which we see as a formidable workflow editor, and the *Grid and User Support Environment*, a web-based framework able to interact with several high-performance computing resources. We have thus created a free and highly accessible way to design workflows on a desktop computer and execute them on high-performance computing resources.

**Conclusions:**

Our work will not only reduce time spent on designing scientific workflows, but also make executing workflows on remote high-performance computing resources more accessible to technically inexperienced users. We strongly believe that our efforts not only decrease the turnaround time to obtain scientific results but also have a positive impact on reproducibility, thus elevating the quality of obtained scientific results.

## Background

The importance of reproducibility for the scientific community has been a topic lately discussed in both high-impact scientific publications and popular news outlets [1, 2]. To be able to independently replicate results—be it for verification purposes or to further advance research—is important for the scientific community. Therefore, it is crucial to structure an experiment in such a way that reproducibility could be easily achieved.

Workflows are structured, abstract recipes that help users construct a series of steps in an organized way. Each step is a parametrised specific action that receives some input and produces some output. The collective execution of these steps is seen as a domain-specific task.

With the availability of biological *big data*, the need to represent workflows in computing languages has also increased [3]. Scientific tasks such as genome comparison, mass spectrometry analysis, protein-protein interaction, just to name a few, access extensive datasets. Currently, a vast number of workflow engines exist [4–8] and each of these technologies has amassed a considerable user base. These engines support, in some way or another, the execution of workflows on distributed high-performance computing (HPC) resources (e.g., grids, clusters, clouds, etc.), thus allowing speedier obtention of results. A wise selection of a workflow engine will shorten the time spent between workflow design and retrieval of results.

### Workflow engines

Galaxy [6] is a free web-based workflow system with several pre-installed tools for data-intensive biomedical research. Inclusion of arbitrary tools is reduced to the trivial task of creating *ToolConfig* [9] files, which are Extensible Markup Language documents (XML). The Galaxy project also features a so-called *toolshed* [10], from which tools can be obtained and installed on Galaxy instances. At the time of writing Galaxy’s *toolshed* featured 3470 tools. However, we have found that Galaxy lacks extended support for popular workload managers and middlewares.

Taverna [7] offers an open-source and domain-independent suite of tools used to design and execute scientific workflows, helping users to automate and pipeline processing of data coming from different web services. At the time of writing Taverna features more than 3500 services available on startup and it also provides access to local and remote tools. Taverna allows users to track results and data flows with great granularity, since it implements the Open Provenance Model standard (OPM) [11]. A very attractive feature of Taverna is the ability to share workflows via the myExperiment research environment [12].

The Konstanz Information Miner Analytics Platform (KNIME Analytics Platform) [4, 13] is a royalty-free engine that allows users to build and execute workflows using a powerful and user-friendly interface. The KNIME Analytics Platform comes preloaded with several ready-to-use tasks (called *KNIME nodes*) that serve as the building stones of a workflow. It is also possible to extend the KNIME Analytics Platform by either downloading community nodes or building custom nodes using a well-documented process [14, 15]. Workflows executed on the KNIME Analytics Platform are limited to run on the same personal computer on which it has been installed, thus rendering it unsuitable for tasks with high-memory or high-performance requirements. KNIME is offered in two variants able to execute workflows on distributed HPC resources: KNIME Cluster Execution [16], and KNIME Server [17]. These two suites are, however, royalty-based—an aspect that might shy away users of the scientific community.

**Fig. 1:**
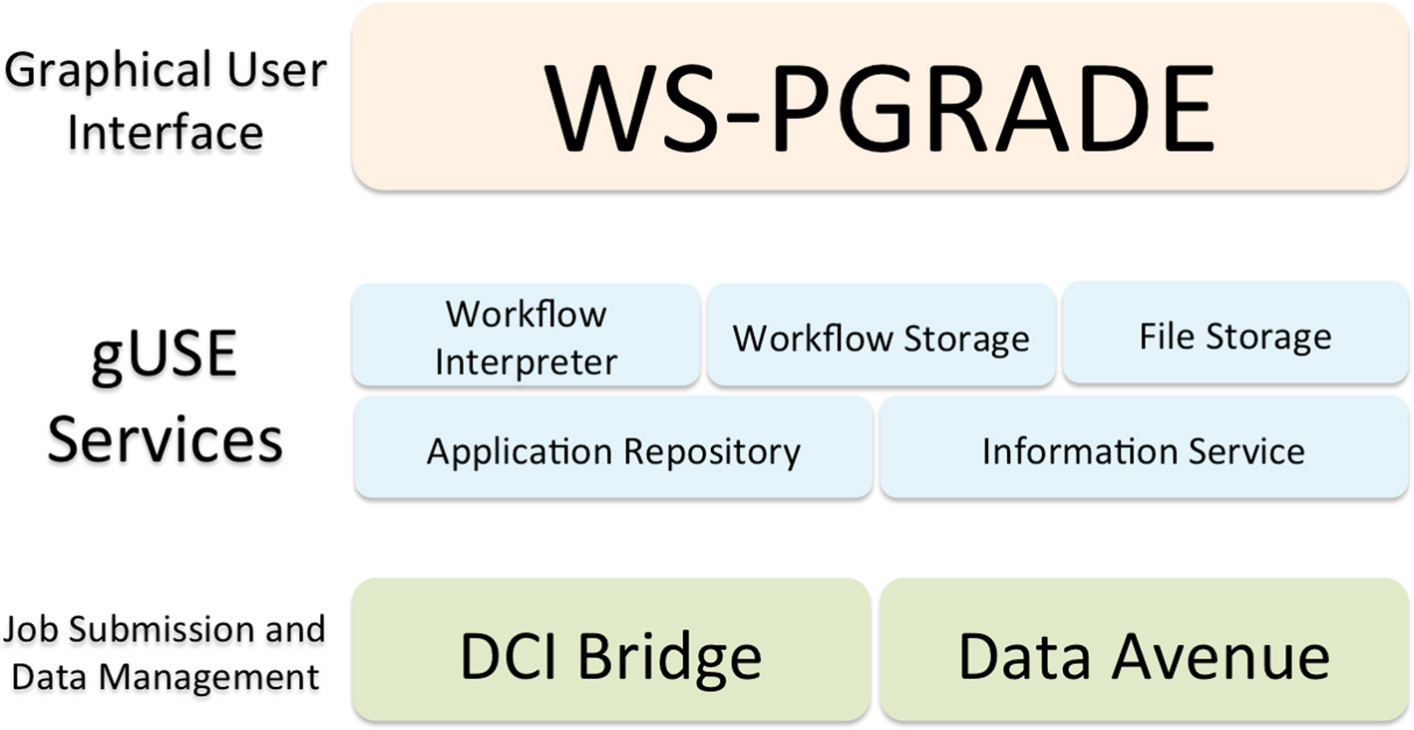
The three-thiered gUSE’s architecture. The three tiers of gUSE’s architecture: WS-PGRADE acts as the user interface, the service layer handles e.g., file, workflow storage. The Job Submission and Data Management layer contains the *DCI Bridge*, which is responsible to access DCIs. Figure based on [47]

The grid and cloud User Support Environment (gUSE) offers an open-source, free, web-based workflow platform able to tap into distributed HPC infrastructures [5]. gUSE entails a set of components and services that offers access to distributed computing interfaces (DCI). The Web Services Parallel Grid Runtime and Developer Environment Portal (WS-PGRADE) component acts as the graphical user interface. This web-based portal is a series of dynamically generated web pages, through which users can create, execute, and monitor workflows. WS-PGRADE communicates with internal gUSE services (e.g., *Workflow Interpreter*, *Workflow Storage*, *Information Service*) using the Web Services Description Language (WSDL) [18]. Passing documents in the WSDL format between its components allows gUSE services to interact with other workflow systems. Figure 1 shows the three-tiered architecture of gUSE. This complex and granular architecture of gUSE enables administrators to distribute the installation of gUSE across resources. A typical set-up is to install WS-PGRADE on a dedicated web server, while installing other services and components on more powerful computers.

**Fig. 2:**
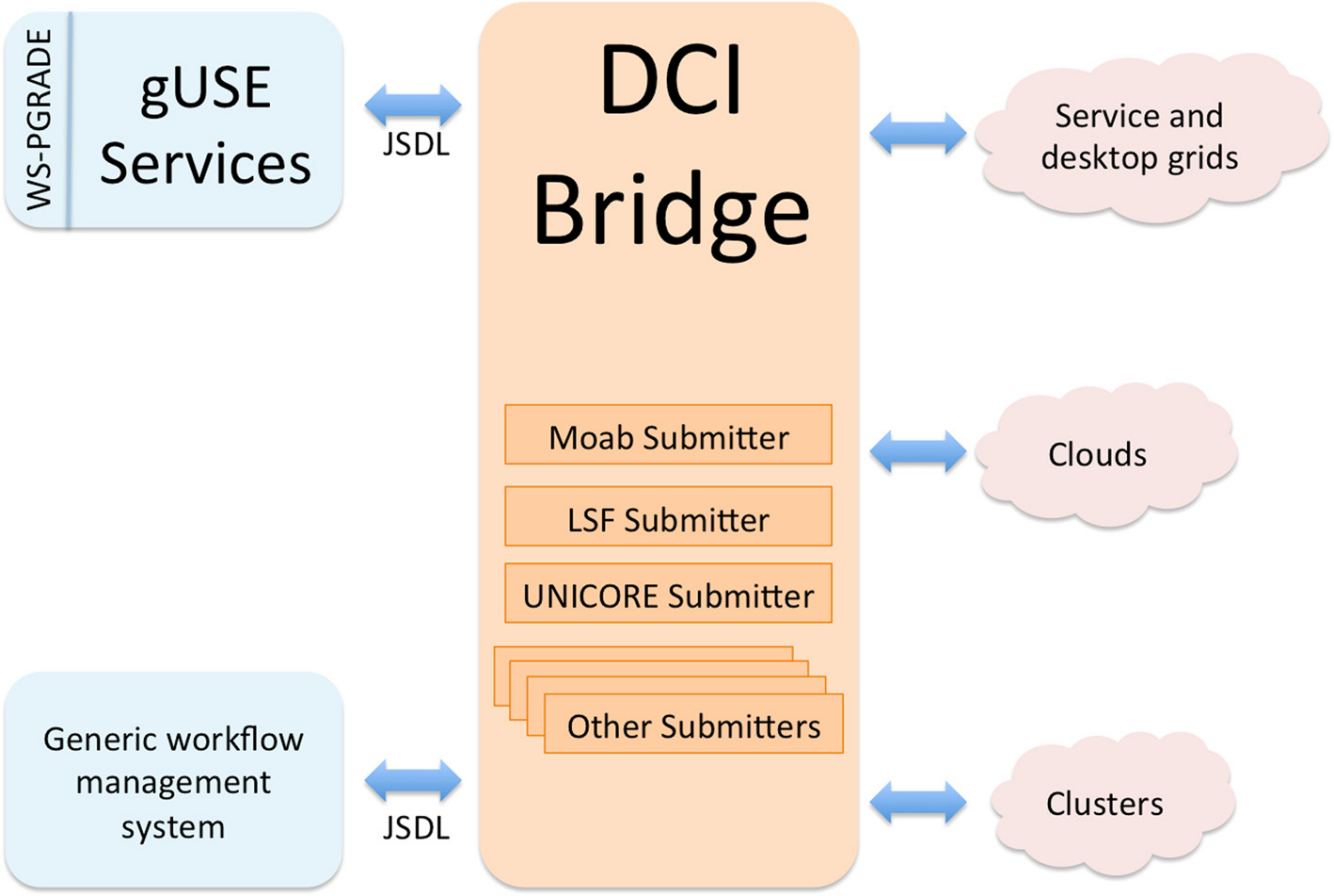
Schematic overview of gUSE’s *DCI Bridge*. Interaction of the *DCI Bridge* with gUSE services and other workflow management systems is done via JSDL requests. The *DCI Bridge* contains *DCI Submitters*, which contain specific code for each of the supported DCIs in gUSE. Figure based on [19]

In order to provide a common workflow submission Application Programming Interface (API), gUSE channels workflow-related requests (i.e., start, monitor, cancel a job on a DCI) through the *DCI Bridge* component [19]. The *DCI Bridge* is fully compatible with the Job Submission Description Language (JSDL) [20], thus enabling other workflow management systems to interact with it in order to benefit from gUSE’s flexibility and DCI support. The *DCI Bridge* contains so-called *DCI Submitters*, each containing specific code to submit, monitor, cancel jobs on each of the supported DCIs (e.g., UNICORE [21], LSF [22], Moab [23]). Figure 2 presents a schematic overview of the interaction between the *DCI Bridge* and other components.

Designing a workflow in gUSE entails two steps: creation of an *abstract graph*, and a node-per-node configuration of the *concrete*. Creation of the *abstract graph* is achieved via a Java WebStart [24] application that is launched from WS-PGRADE, but is executed on the user’s computer. At this point, users are able to provide only application domain information. After saving the *abstract graph*, users would direct their web browser back to WS-PGRADE, open the *concrete* for editing, and configure each task comprising the workflow separately. The configuration entails details such as provision of the required command line arguments to execute each task. Since gUSE offers interfaces to several middlewares, it is possible to execute tasks of the same workflow on different DCIs. The possible complexity of workflows that could be executed by gUSE is reflected in the several available fields on the task configuration windows presented to the user while configuring the *concrete* (see Fig. 6). The two-step creation of workflows (i.e., creation of the *abstract graph* and creation/configuration of the *concrete*, as depicted in Fig. 6), combined with the steep learning curve that gUSE poses to new users is an aspect that might intimidate users without much technical experience.

Due to the diversity of workflow engines, a variety of workflow representations has arisen. This disparity of representations poses a challenge to scientists who desire to reuse workflows. Ideally, a scientist would design and test a workflow only once and it would be possible to execute it on any workflow engine on a given DCI. Built on this principle, the Sharing Interoperable Workflow for Large-Scale Scientific Simulation on Available DCIs project [25] (SHIWA) allows users to run previously existing workflows from different platforms on the SHIWA Simulation Platform. However, privacy concerns might give scientists second thoughts about executing their workflows and uploading their sensitive data on the SHIWA Simulation Platform. Tavaxy [8], focusing on genome comparison and sequence analysis, was created to enable the design of workflows composed of Taverna and Galaxy sub-workflows, and other workflow nodes in a single environment.

There is a clear overlap between the functionalities of the mentioned workflow engines. All offer royalty-free workflow design and execution. However, based on feedback from users and experience in our research group, we believe that the KNIME Analytics Platform is an accessible workflow editor, although it lacks on computing power. On the other hand, again based on experience and feedback, we see gUSE as a great *back end* framework that taps into several DCIs, but we have found that its workflow editing capabilities pose a steep learning curve to its user base.

In this paper we present work that bridges the gap between the KNIME Analytics Platform and gUSE. Our work will surely help adopters to significantly reduce the time spent designing, creating and executing repeatable workflows on distributed HPC resources.

### Workflow representation

#### Formal representation

Workflows can be represented using Petri nets [26, 27]. Petri nets are directed bipartite graphs containing two kinds of nodes: *places* and *transitions*.

A *place* represents a set of conditions that must be fulfilled for a *transition* to occur. Once a *place* has satisfied all its conditions, it is *enabled*. *Transitions* represent actions that affect the state of the system (i.e., copy a file, perform a computation, modify a file).

*Places* and *transitions* are connected by edges called *arcs*. No *arc* connects two nodes of the same kind (i.e., this restriction is precisely what makes Petri nets bipartite graphs). *Arcs* represent *transitions*’ pre- and postconditions. In order for a *transition* to take place, all of its preceding *places* must be *enabled* (i.e., all of the conditions of preceding *places* must be satisfied). Conversely, when a *transition* has been completed, a postcondition is satisfied, influencing the state of subsequent *places* to which this *transition* is connected to.

**Fig. 3:**
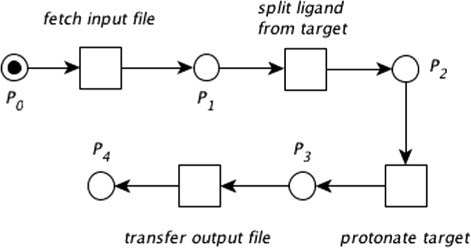
A workflow as represented by a Petri net. Petri net modelling a software pipeline to protonate a molecule found in a single input file. *Places* are shown as circles, *transitions* are depicted as squares. The *place* *P*_0_ expects and contains one *token*, represented by a black dot, and is thus *enabled*. It follows that *P*_0_ is the starting *place* and *P*_4_ represents the end of the process

Whenever a *place*’s condition is satisfied, a *token* is depicted inside the corresponding *place*. Figure 3 depicts a simple computer process in which a molecule contained in an input file will be modified by adding any missing hydrogen atoms (i.e., the molecule will be protonated).

#### High-level representation

There are several alternatives to represent workflows in a platform-independent way. Yet another Workflow Language (YAWL) [28] was created after extensive analysis and review of already existing workflow languages in order to identify common workflow patterns and develop a new language that could combine the strengths and overcome the handicaps of other languages. The Interoperable Workflow Intermediate Representation (IWIR) [29] is a standard adopted by several workflow engines and is the language describing workflows in the SHIWA Simulation Platform [25]. More recently a group of individuals, vendors and organizations joined efforts to create the Common Workflow Language (CWL) [30] in order to provide a specification that enables scientists to represent workflows for data-intensive scientific tasks (e.g., mass spectrometry analysis, sequence analysis).

For the sake of clarity and brevity, literature commonly depicts workflows as directed acyclical graphs, in which each vertex represents a *place* together with its pre- and postconditions (i.e., the preceding and following *transitions*) [31]. Each of the vertices is labelled, has a unique identifier and represents a task to be performed. Furthermore, each of the tasks in a workflow can receive inputs and can produce outputs. Outputs of a task can be channeled through another task as an input. An edge between two nodes represents the channeling of an output from a task into another. Edges determine the logical sequence to be followed (i.e., the *origin* task of an edge has to be completed before the *destination* task of an edge can be performed). A task will be executed once all of its inputs can be resolved. Workflow tasks are commonly referred by workflow systems as *nodes* or *jobs* and in this manuscript we will use these terms interchangeably.

### Workflow abstraction

Workflows contain three different dimensions or abstraction layers, namely, *case*, *process* and *resource* dimensions [26]. Mapping these dimensions into concepts used on distributed execution workflow systems, we find that:

- The *case* dimension refers to the execution of a workflow (i.e., a single *run*).
- The *process* dimension, also referred to as the *abstract* layer [32, 33], deals with the application domain (i.e., the purpose of the workflow), therefore, technical details such as architecture, platform, libraries, implementation, programming language, etc., are hidden in this dimension.
- The *resource* dimension, often called the *concrete* layer [32, 33], encompasses the hardware and software used to execute the desired process; questions such as *How many processors does a task require?*, *Which software running on which device will perform a certain task?*, *What are the provided parameters for a specific task?*, etc., must be answered in this layer.

**Fig. 4:**
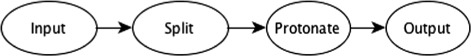
An *abstract* workflow. Each vertex corresponds to a task and each edge corresponds to the logical sequence to be followed. Only application domain information is present

Given that the focus of our work is distributed workflows, we prefer the use of the *abstract*/*concrete* terminology throughout this document. Figure 4 depicts the *abstract* layer of the previously introduced protonation process. This workflow is now composed of four tasks (i.e., vertices) and three edges. The task labelled *Input* has no predecessor tasks, therefore this task is the first one to be executed. In comparison, the task labelled *Protonate* depends on the completion of *Split*, which in turn depends on the completion of *Input*.

**Fig. 5:**
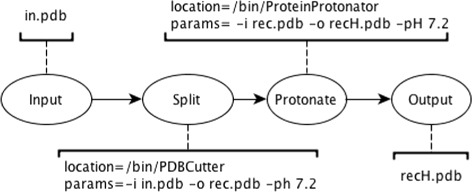
A *concrete* workflow. Similar to an *abstract* workflow, a *concrete* workflow contains implicit application domain information. However, vertices of *concrete* workflows are annotated with extra attributes needed to *actually* execute the given tasks and obtain the needed data

Figure 5, in contrast to Fig. 4, shows a possible *concrete* representation of the presented sample workflow, in which each vertex has been annotated with information needed in order to actually execute the corresponding tasks. While this information varies across workflow execution systems and platforms, the *abstract* representation of a workflow is constrained only to the application domain and is thus independent of the underlying software and infrastructure. At the *concrete* layer, the edges not only determine the logical sequence to be followed, but also represent channeled output files. For instance, the *Protonate* task receives an input file from its predecessor, *Split* and generates an output file that will be channeled to the *Output* task.

**Fig. 6:**
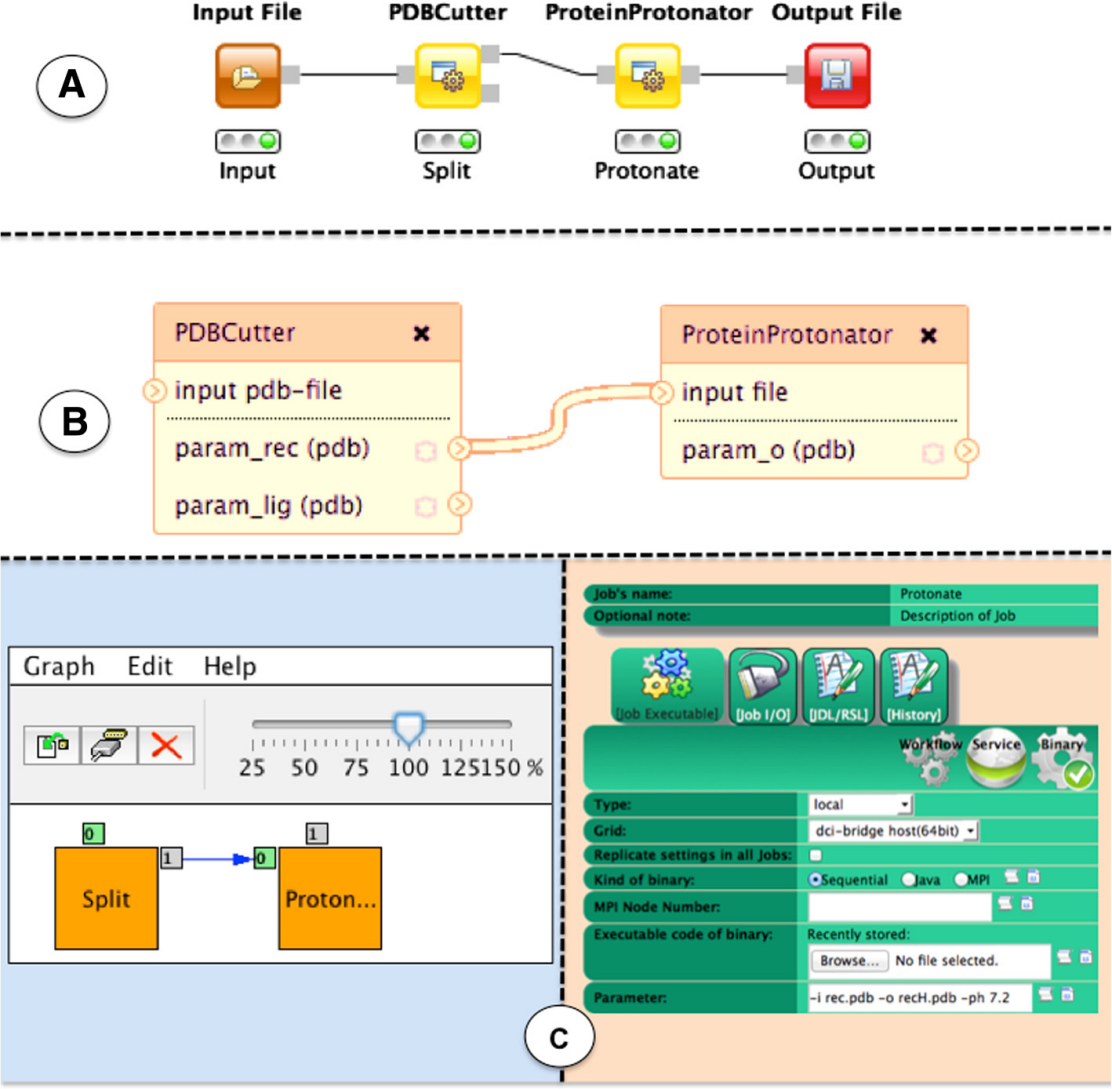
Comparison of the same pipeline across KNIME Analytics Platform, Galaxy, gUSE. The KNIME Analytics Platform and Galaxy (sections A, B, respectively) offer an intuitive workflow creation and there is no perceived boundary between the *abstract* and the *concrete* layers. gUSE, however, (section C) splits the creation of workflows in two phases, creation of the *abstract* graph and the further configuration of each node in the *concrete* workflow

### Workflow conversion

As it has been mentioned before, gUSE splits the creation of workflows into two steps, creation of the *abstract graph* and configuration of the *concrete*. These steps match the *abstract* and *concrete* layers we have discussed in the previous section. Both Galaxy and the KNIME Analytics Platform, in turn, represent workflows without this separation. In order to create a workflow in either Galaxy or the KNIME Analytics Platform, users *drag and drop* visual elements representing nodes into a *workspace*. Nodes come pre-configured and commonly execute a single task, therefore the user creates both the *abstract* and *concrete* layer at the same time. Inputs and outputs of nodes are visually represented and they can be connected to other nodes. Each node has a configuration dialog in which users can change the default parameters.

It is easy to see how functionally equivalent workflow constructs (e.g., conditional execution of a workflow section, parameter sweep, etc.) are represented differently across workflow engines. Furthermore, engines may offer features that are not available on other workflow systems (e.g., the KNIME Analytics Platform offers elements that are not present neither in gUSE nor in Galaxy, such as *flow variables* [34]). The proper identification and conversion of these elements is important for the conversion, since they play their part in workflow execution. Figure 6 displays a schematic comparison of the implementation of our example workflow across three selected workflow engines, the KNIME Analytics Platform, Galaxy, and gUSE.

Due to the variety of workflow systems—we have mentioned only a few instances, it is not surprising that there are several languages and file formats to represent workflows. A first step towards a successful conversion of workflows is to be able to represent the vertices of a workflow (i.e., the tasks, or nodes) in a consistent way across platforms. Once a platform-independent representation of the vertices has been achieved, it is easier to import tasks into several workflow engines with less effort. Conversion of edges is an endeavour that is specific to each of the workflow engines (i.e., a task can be seen as a standalone component, while edges are the result of the collaboration of two or more tasks). Over the course of the last years, we have developed a series of tools enabling workflow interoperability across disparate workflow engines.

## Implementation

Conversion of whole workflows can be split into two parts, namely, conversion of vertices and conversion of edges. Vertices represent tasks that take inputs, parameters and produce outputs. This information can be represented in a structured way.

**Fig. 7:**
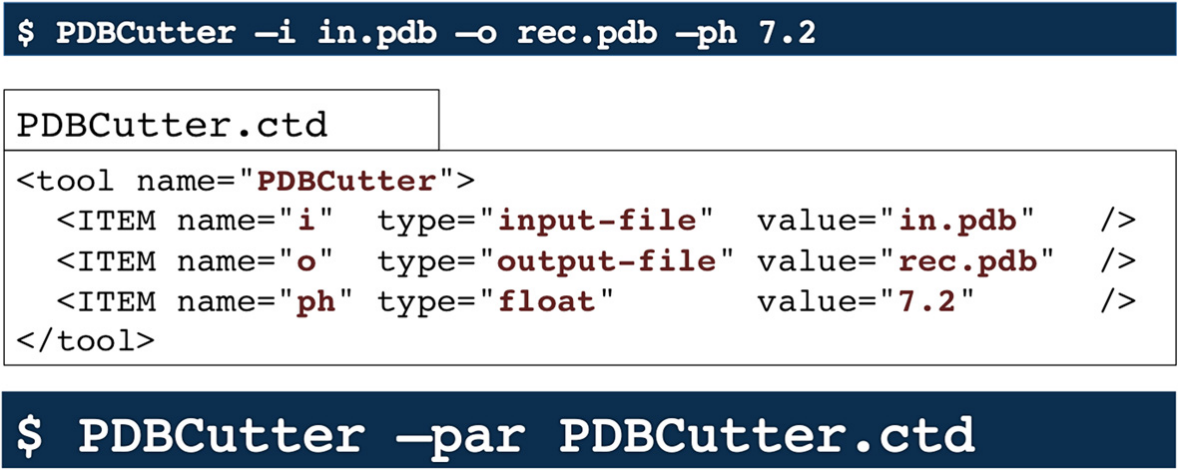
A CTD in action. The upper section shows all three parameters needed for the tool *PDBCutter* to be executed. The middle section shows a snippet of a CTD representing a *CTD-enabled* tool. The bottom section shows how to execute a *CTD-enabled* tool with the given sample CTD

**Fig. 8:**
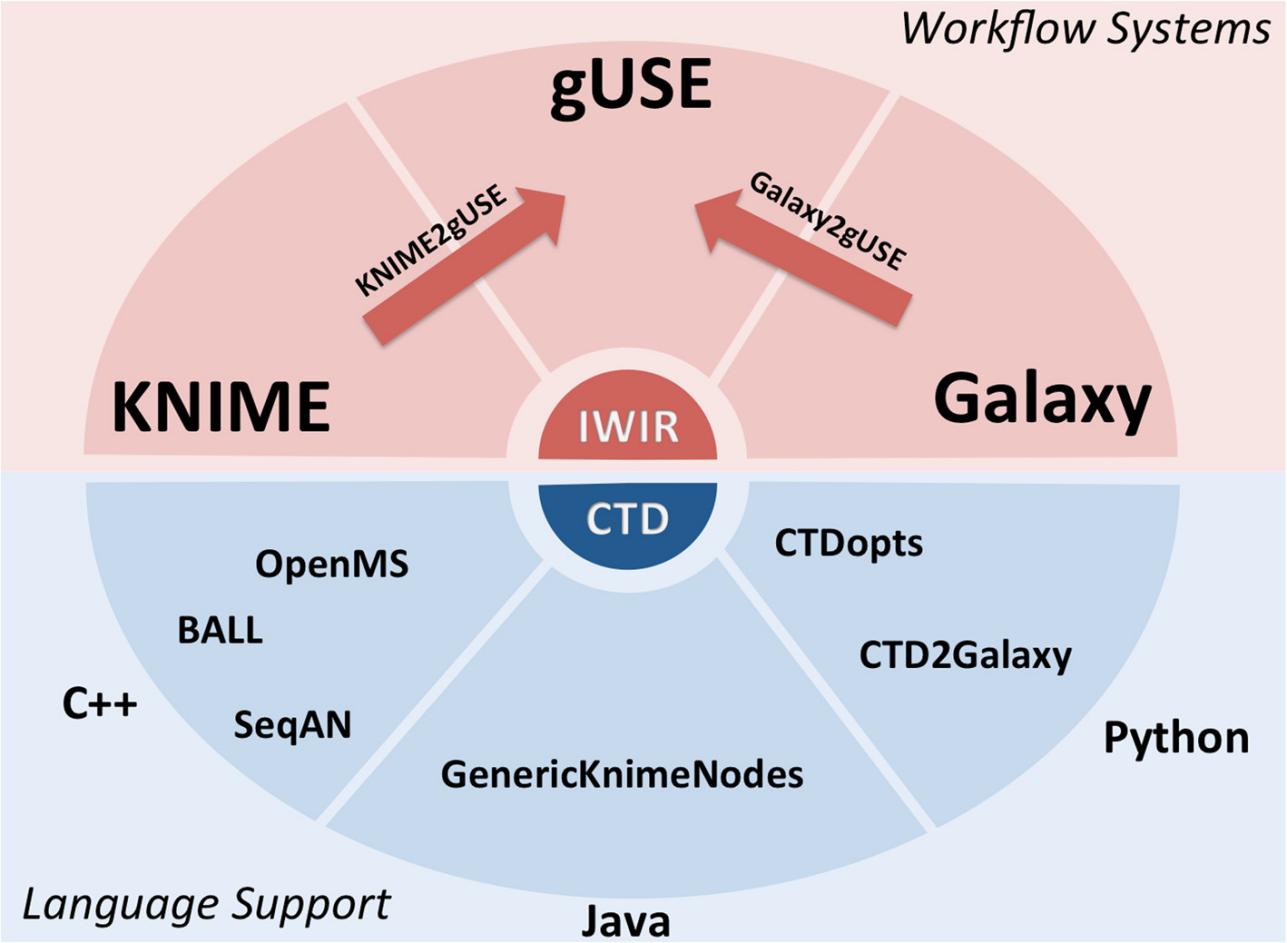
Overview of how CTDs interact with programming languages and workflow systems. CTDs can be generated by *CTD-enabled* tools (e.g., BALL, OpenMS, SeqAn) or via CTDopts. Once a tool is *CTD-enabled*, it can be imported into the KNIME Analytics Platform or Galaxy. We have also developed converters that can import KNIME Analytics Platform, Galaxy workflows into gUSE to take advantage of HPC resources and DCIs

Common Tool Descriptor files (CTD) are XML documents that contain information about parameters, inputs and outputs of a given tool. This information is presented in a structured and human readable way, thus facilitating manual generation for arbitrary tools. Since CTDs are also properly formatted XML documents, it is a trivial matter to parse them in an automated way. Generation of CTDs can be either done manually or by *CTD-enabled* programs. Software libraries and suites such as SeqAn [35], OpenMS [36] and BALL [37] are *CTD-enabled*, that is, they are able to generate CTDs for each of its tools, parse input CTDs and execute tools accordingly. Executing a *CTD-enabled* tool across different platforms is a process transparent for end users. Figures 7 and 8 display how CTDs encapsulate needed runtime information into a single file, and how CTDs interact with other languages and platforms, respectively.

We have also worked towards making CTDs more accessible. Taking into account that refactoring tools to make them *CTD-enabled* might be time consuming, we have developed CTDopts in order to bind an already existing tool via Python wrappers. Naturally, this interaction is not done automatically without any further input, but this is an easier endeavor than performing a refactoring. CTDopts acts as a wrapper allowing users to execute arbitrary command line tools via CTDs. These Python wrappers will communicate directly with the tool, thus offering end users an interface to a *CTD-enabled* tool. Of course, manual creation of CTDs is always an option. Since CTDs are XML documents, a text editor is all is needed to manually generate them.

Representing a simple vertex using CTDs brings users closer to workflow interoperability, but this exercise might not pay off on its own. In order to extend a workflow engine by adding new tools, one could delve into the inner-workings of said workflow engine and import new tools. Since this could be a technical effort not accessible to users without the needed experience, we have also developed converters of tasks (i.e., vertices).

The KNIME Analytics Platform is an application based on the Eclipse Modelling Framework (EMF) [38], allowing the development of extensions. One of these extensions, and perhaps the most interesting for KNIME Analytics Platform users, is the ability to develop new *KNIME nodes* [14]. It is also possible to download so-called *community nodes* [15].

We developed the Generic KNIME Nodes (GKN) extension to make use of KNIME Analytics Platform’s extensibility. GKN takes a set of CTDs as an input and generates the needed resources to implement *KNIME nodes*. In order to achieve this, its functionality is split into two main components: *node generation* and *node execution*. Once a node built via GKN has been generated (i.e., via the *node generation* component) and imported into the KNIME Analytics Platform, it interacts with other *KNIME nodes* via the *node execution* component. This interaction is transparent for the user.

Since Galaxy is one of the most popular workflow systems in the bioinformatics community, we felt that providing a suitable conversion would benefit the scientific community, so we developed CTD2Galaxy, a set of scripts to convert a CTD into a Galaxy *ToolConfig* XML file [9]. We also analysed Galaxy’s *toolshed* [10] and we determined that it would be possible to automatically convert around 1200 of these tool descriptions into CTD files. The rest of the tools in the *toolshed* contain elements that are not easily translated and are not supported in CTD format (e.g., *if-else* constructs, *for loops*, etc.).

So far, we have discussed conversion of vertices. Different workflow engines represent workflows using a different format. It follows that conversion of edges is an effort that heavily depends on the involved workflow engines. Analog to the need of a platform-independent representation of vertices, a first step of workflow interoperability, which is our end target, is the development of a platform-independent representation of workflows.

In spite of the apparent variety of workflow languages, we have focused our efforts in using the KNIME Analytics Platform as the source point of workflow conversion. To this end, we have also started work that could translate *any* KNIME Analytics Platform workflow into either an IWIR representation or a gUSE workflow (KNIME2gUSE). Alternatively, we have also developed a set of scripts that convert Galaxy workflows into gUSE (Galaxy2gUSE). Refer to Fig. 8 for a brief overview of how our work fits together against workflow systems.

## Results and discussion

We have implemented a Label-free-quantification (LFQ) [39, 40] workflow in the KNIME Analytics Platform. LFQ is a widely used type of experiment in mass spectrometry based proteomics aimed at quantifying and comparing the abundances of peptides and proteins across different samples. Unlike other quantification strategies employing various kinds of chemical labelling of the different samples, LFQ does not impose a limit on the number of samples. Experiments with tens or hundreds of samples are routinely performed in many labs and, considering the ever-increasing performance of modern mass spectrometers, the number of samples to be analyzed per experiment is very likely to keep growing. This, in turn, gives rise to major computational challenges when analyzing the resulting large and complex data sets consisting of up to several terabytes of raw data. Hence, data processing and analysis of label-free quantification experiments can greatly benefit from distributed HPC resources and shall therefore serve as an example use case.

Our example workflow is based on tools provided by OpenMS/TOPP [36, 41–43]. In addition to label-free quantification, it performs a complete consensus peptide identification [44] using the search engines OMSSA [45] and X!Tandem [46]. In essence, so-called tandem mass spectra containing the masses of fragment ions resulting from collision-induced dissociation of selected peptides are compared to theoretical fragment spectra generated from a given FASTA database containing protein sequences. Afterwards, peptide hits are filtered so that the remaining set of identifications has a false discovery rate of less than 1 %. The quantification part starts with two major steps of data reduction and signal detection: peak picking and feature finding. Subsequently, the results of the identification and quantification branches of the workflow are combined, and corresponding peptide signals are matched across all samples in a process called feature linking. Finally, a normalization step is performed, which is necessary in order to be able to actually compare the relative abundances of peptides across the different runs. It is important to note that each run is executed independently via parameter sweep. Furthermore, each run is represented in a different input file, as given by the *Input Files* element. The output of the complete workflow is channeled to the *TextExporter* tool, which in turn generates a single comma-separated values (CSV) file containing all identified peptides together with their abundances in all given samples.

**Fig. 9:**
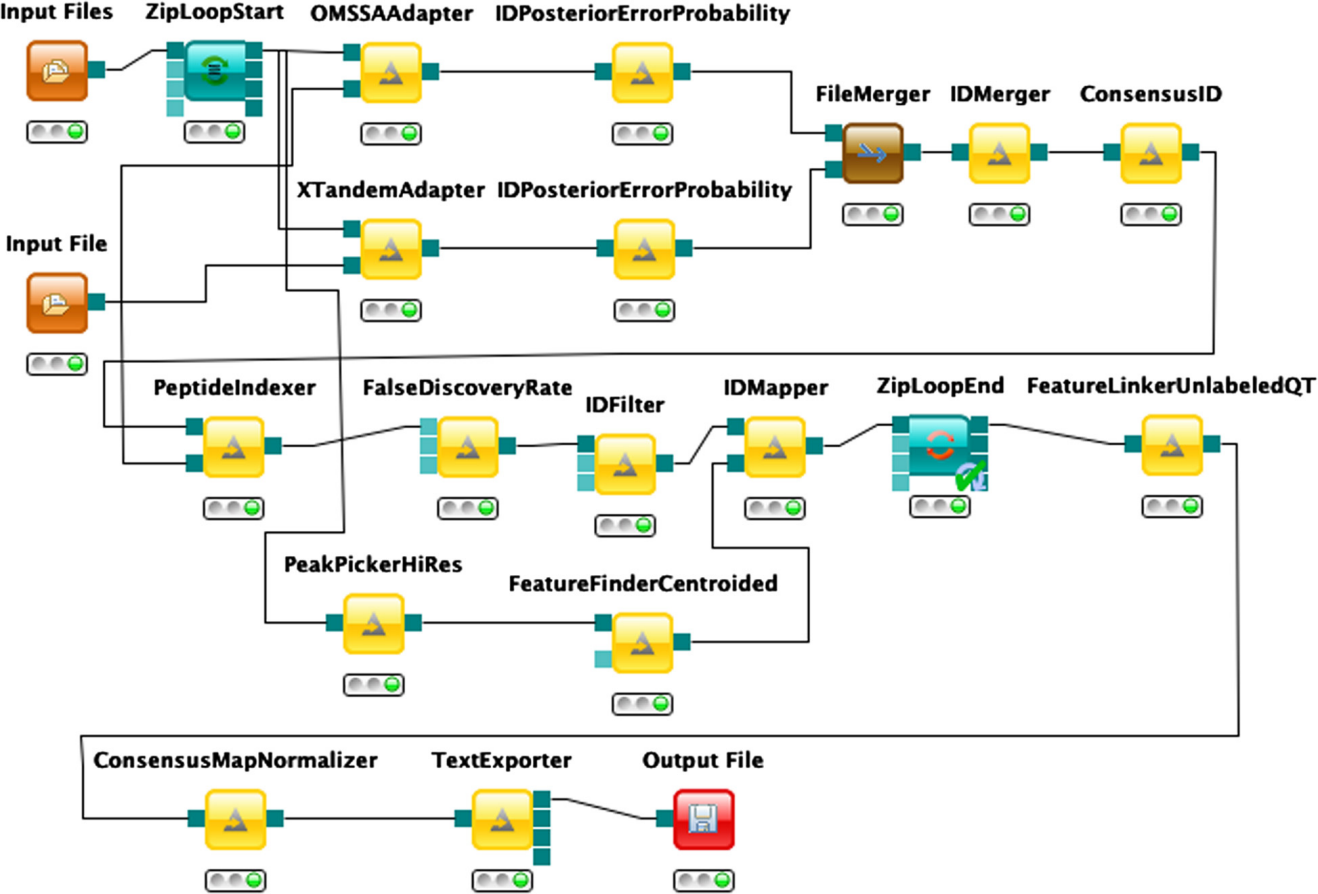
Label-free Quantification pipeline implemented in the KNIME Analytics Platform. The section enclosed by the *ZipLoopStart* and *ZipLoopEnd* will be executed independently for each of the given input files (i.e., parameter sweep)

**Fig. 10:**
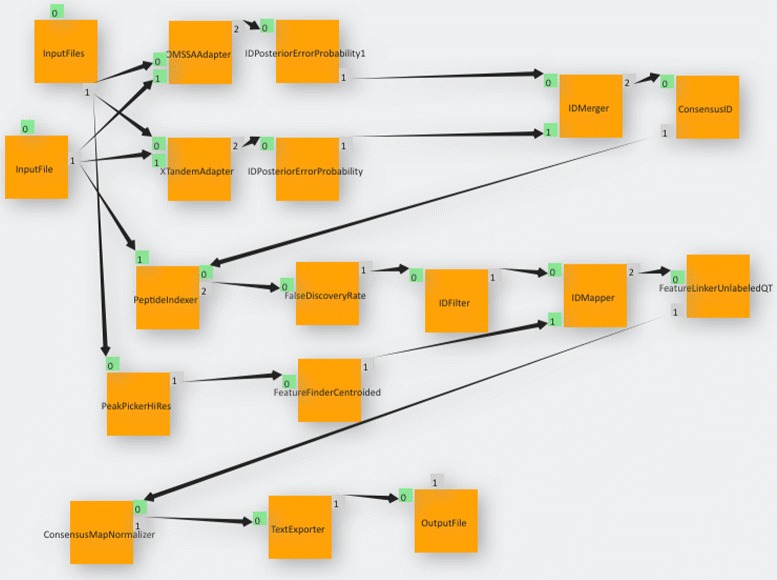
Label-free Quantification pipeline as imported from the KNIME Analytics Platform into gUSE using the KNIME2gUSE extension. Note how parameter seep elements depicted in Fig. 9 such as *ZipLoopStart* and *ZipLoopEnd* are not present in gUSE. This is due to the fact that gUSE implements parameter seep by setting properties in input and output ports of the corresponding nodes

Figure 9 depicts the implementation of our LFQ workflow. We used our KNIME2gUSE extension and successfully imported our workflow in gUSE, as Fig. 10 shows.

## Conclusions

Throughout this document we have presented our work towards workflow interoperability. We are convinced that investing time and effort in workflow interoperability helps scientists from all fields to expedite retrieval of results, so we tested and analyzed several workflow engines.

Based on user feedback and our own usage experience, we noticed that the creation of workflows in the KNIME Analytics Platform is straightforward, rapid and user-friendly. The needed amount of previous knowledge of the KNIME Analytics Platform or other workflow systems to put together a workflow and execute it is minimal. However, we were not satisfied with the fact that execution of workflows on distributed HPC resources is royalty-based. Our search then brought us to gUSE.

gUSE is an open-source web-based framework that enables users to execute workflows on distributed HPC resources. It supports several major resource managers and middlewares via the use of so-called *DCI Submitters*, which can also be added to extend gUSE’s support. However, workflow creation in gUSE is not as straightforward as in the KNIME Analytics Platform.

It was apparent that there was a need for a solution that combined the features and overcame the drawbacks of these two framework systems. On one side, a free and easy-to-use workflow editor and on the other side, a free and powerful back-end system connecting to several distributed HPC resources.

We are confident that our work presented in this document, in particular KNIME2gUSE, not only provides scientists a way to design and test workflows on their desktop computers, but also enables them to use powerful resources to execute their workflows, thus producing scientific results in a timely manner. We see KNIME2gUSE as a potential adopter of CWL: KNIME2gUSE could be extended in order to generate a CWL representation of a KNIME Analytics Platform workflow.

## Availability and requirements

- **Project name:** Workflow Conversion.
- **Project home page:**http://workflowconversion.github.io/
- **Operating system(s):** Platform independent.
- **Programming language:** Java, Python.
- **Other requirements:** e.g. Python 2.7, Java 1.6 or higher.
- **License:** e.g. GNU General Public License (GPL).
- **Any restrictions to use by non-academics:** none.

## Abbreviations

API: Application programming interface
BALL: Bioinformatics algorithms library
CSV: Comma separated values
CTD: Common tool descriptor
CWL: Common workflow language
DCI: Distributed computing infrastructure
EMF: Eclipse modelling framework
GKN: Generic KNIME nodes
gUSE: Grid and cloud user support environment
HPC: High-performance computing
IWIR: Interoperable workflow intermediate representation
JSDL: Job submission description language
KNIME: Konstanz information miner
LFQ: Label-ree quantification
LSF: Platform load sharing facility
OPM: Open provenance model
OMSSA: Open mass spectrometry search algorithm
SHIWA: Sharing interoperable workflow for large-scale scientific simulation on available DCIs project
TOPP: The OpenMS proteomics pipeline
UNICORE: Uniform interface to computing resources
WSDL: Web services description language
WS-PGRADE: Web services parallel grid runtime and developer environment portal
XML: Extensible markup language
YAWL: Yet another Workflow Language

## References

[1] Gratzer W (2013). Trouble at the lab. Economist.

[2] McNutt M (2014). Reproducibility. Science (New York, N.Y.).

[3] Greene CS, Tan J, Ung M, Moore JH, Cheng C (2014). Big data bioinformatics. J Cell Physiol.

[4] Berthold MR, Cebron N, Dill F, Gabriel TR, Kotter T, Meinl T, Ohl P, Sieb C, Thiel K, Wiswedel B. Knime. Web. 2007:1–8. doi:http://dx.doi.org/10.1007/978-3-540-78246-9.

[5] Kacsuk P, Farkas Z, Kozlovszky M, Hermann G, Balasko A, Karoczkai K, Marton I (2012). WS-PGRADE/gUSE generic DCI gateway framework for a large variety of user communities. J Grid Comput.

[6] Blankenberg D, Kuster GV, Coraor N, Ananda G, Lazarus R, Mangan M, Nekrutenko A, Taylor J. Galaxy: A web-based genome analysis tool for experimentalists. 2010. http://arxiv.org/abs/NIHMS150003 doi:http://dx.doi.org/10.1002/0471142727.mb1910s89 10.1002/0471142727.mb1910s89 PMC4264107 20069535

[7] Missier P, Soiland-Reyes S, Owen S, Tan W, Nenadic A, Dunlop I, Williams A, Oinn T, Goble C. Taverna, reloaded. In: Lecture Notes in Computer Science (including Subseries Lecture Notes in Artificial Intelligence and Lecture Notes in Bioinformatics), vol. 6187 LNCS: 2010. p. 471–81, doi:http://dx.doi.org/10.1007/978-3-642-13818-8_33.

[8] Abouelhoda M, Issa S, Ghanem M. Tavaxy: Integrating Taverna and Galaxy workflows with cloud computing support. 2012. doi:http://dx.doi.org/10.1186/1471-2105-13-77. 10.1186/1471-2105-13-77 PMC3583125 22559942

[9] Galaxy Tool XML File. https://wiki.galaxyproject.org/Admin/Tools/ToolConfigSyntax. Accessed 28 July 2015.

[10] Galaxy Tool Shed. https://toolshed.g2.bx.psu.edu/. Accessed 07 July 2015.

[11] Moreau L, Clifford B, Freire J, Futrelle J, Gil Y, Groth P, Kwasnikowska N, Miles S, Missier P, Myers J, Plale B, Simmhan Y, Stephan E, Den Bussche JV. The Open Provenance Model core specification (v1.1). In: Future Generation Computer Systems, vol. 27: 2011. p. 743–56, doi:http://dx.doi.org/10.1016/j.future.2010.07.005.

[12] Goble CA, Bhagat J, Aleksejevs S, Cruickshank D, Michaelides D, Newman D, Borkum M, Bechhofer S, Roos M, Li P, de Roure D. myExperiment: A repository and social network for the sharing of bioinformatics workflows. Nucleic Acids Res. 2010;38(SUPPL. 2). doi:http://dx.doi.org/10.1093/nar/gkq429. 10.1093/nar/gkq429 PMC2896080 20501605

[13] KNIME | Open for Innovation. http://www.knime.org/. Accessed 29 June 2015.

[14] KNIME | New Node Wizard. https://tech.knime.org/new-node-wizard. Accessed 06 July 2015.

[15] KNIME | Community Contributions. https://tech.knime.org/community. Accessed 07 July 2015.

[16] KNIME | KNIME Cluster Execution. https://www.knime.org/cluster-execution. Accessed 06 July 2015.

[17] KNIME | KNIME Server - The Heart of a Collaborative KNIME Setup. https://www.knime.org/knime-server. Accessed 06 July 2015.

[18] Web Service Definition Language (WSDL). http://www.w3.org/TR/wsdl. Accessed 06 July 2015.

[19] DCI Administration Manual, Version 3.7.1. http://sourceforge.net/projects/guse/files/3.7.1/Documentation/DCI_BRIDGE_MANUAL_v3.7.1.pdf/download.

[20] Anjomshoaa A, Brisard F, Drescher M, Fellows D, Ly A, McGough S, Pulsipher D, Savva A. Job Submission Description Language (JSDL) Specification, Version 1.0. 2005:1–72. Open Grid Forum.

[21] Romberg M (2002). The UNICORE Grid Infrastructure. Spec Issue Grid Comput Scientifc Program J.

[22] IBM Platform Computing Products: Workload Management Platform - Platform LSF. IBM Corporation. 2012. http://www-03.ibm.com/systems/platformcomputing/products/lsf/.

[23] HPC Products - Adaptive Computing. http://www.adaptivecomputing.com/products/hpc-products/. Accessed 06 July 2015.

[24] Java SE Desktop Technologies - Java Web Start Technology. http://www.oracle.com/technetwork/java/javase/javawebstart/index.html. Accessed 03 July 2015.

[25] Terstyanszky G, Kukla T, Kiss T, Kacsuk P, Balasko A, Farkas Z (2014). Enabling scientific workflow sharing through coarse-grained interoperability. Futur Gener Comput Syst.

[26] van der Aalst WMP. The application of petri nets to workflow management. 1998. doi:http://dx.doi.org/10.1142/S0218126698000043.

[27] Peterson JL, Vol. 24. Petri Net Theory and the Modeling of Systems; 1981, p. 290.

[28] van der Aalst, ter Hofstede AHM (2005). YAWL: yet another workflow language. Inf Syst.

[29] Plankensteiner K, Montagnat J, Prodan R. IWIR: A Language Enabling Portability Across Grid Workflow Systems. In: SIGMOD Rec: 2011. p. 97–106, doi:http://dx.doi.org/10.1145/2110497.2110509. http://doi.acm.org/10.1145/2110497.2110509.

[30] Common Workflow Language. http://www.oracle.com/technetwork/java/javase/javawebstart/index.html. Accessed 03 July 2015.

[31] Salimifard K, Wright M. Petri net-based modelling of workflow systems: An overview. 2001. doi:http://dx.doi.org/10.1016/S0377-2217(00)00292-7.

[32] Deelman E, Blythe J, Gil Y, Kesselman C, Mehta G, Vahi K, Blackburn K, Lazzarini A, Arbree A, Cavanaugh R, Koranda S (2003). Mapping abstract complex workflows onto grid environments. J Grid Comput.

[33] Yu J, Buyya R. A taxonomy of scientific workflow systems for grid computing. 2005. doi:http://dx.doi.org/10.1145/1084805.1084814.

[34] KNIME | Flow Variables. https://tech.knime.org/wiki/flow-variables. Accessed 26 Oct 2015.

[35] Döring A, Weese D, Rausch T, Reinert K (2008). SeqAn an efficient, generic C++ library for sequence analysis. BMC Bioinforma.

[36] Sturm M, Bertsch A, Gröpl C, Hildebrandt A, Hussong R, Lange E, Pfeifer N, Schulz-Trieglaff O, Zerck A, Reinert K, Kohlbacher O (2008). Openms - an open-source software framework for mass spectrometry. BMC Bioinforma.

[37] Hildebrandt A, Dehof AK, Rurainski A, Bertsch A, Schumann M, Toussaint NC, Moll A, Stöckel D, Nickels S, Mueller SC, Lenhof HP, Kohlbacher O (2010). BALL–biochemical algorithms library 1.3. BMC Bioinforma.

[38] Steinberg D, Budinsky F, Paternostro M, Merks E. EMF: Eclipse Modeling Framework; 2008, p. 744.

[39] Bantscheff M, Schirle M, Sweetman G, Rick J, Kuster B (2007). Quantitative mass spectrometry in proteomics: a critical review. Anal Bioanal Chem.

[40] Weisser H, Nahnsen S, Grosman J, Nilse L, Quandt A, Brauer H, Sturm M, Kenar E, Kohlbacher O, Aebersold R, Malmstrom L (2013). An automated pipeline for high-throughput label-free quantitative proteomics. J Proteome Res.

[41] Kohlbacher O, Reinert K, Gröpl C, Lange E, Pfeifer N, Schulz-Trieglaff O, Sturm M (2007). Topp–the openms proteomics pipeline. Bioinformatics.

[42] Junker J, Bielow C, Bertsch A, Sturm M, Reinert K, Kohlbacher O (2012). Toppas: a graphical workflow editor for the analysis of high-throughput proteomics data. J Proteome Res.

[43] OpenMS | An Open-source Framework for Mass Spectrometry and TOPP – The OpenMS Proteomics Pipeline. http://open-ms.sourceforge.net/. Accessed 26 June 2015.

[44] Nahnsen S, Bertsch A, Rahnenführer J, Nordheim A, Kohlbacher O (2011). Probabilistic consensus scoring improves tandem mass spectrometry peptide identification. J Proteome Res.

[45] Geer LY, Markey SP, Kowalak JA, Wagner L, Xu M, Maynard DM, Yang X, Shi W, Bryant SH (2004). Open mass spectrometry search algorithm. J Proteome Res.

[46] Craig R, Beavis RC (2004). Tandem: matching proteins with tandem mass spectra. Bioinformatics.

[47] gUSE in a Nutshell. http://sourceforge.net/projects/guse/files/gUSE_in_a_Nutshell.pdf/download.

